# Research on UAV Flight Parameter Identification Method Based on Launch Force and Airspeed

**DOI:** 10.3390/s24051597

**Published:** 2024-02-29

**Authors:** Zhipeng Chen, Haojie Li, Hang Yu, Yuan Zhao, Chuanhao Zhang, He Zhang

**Affiliations:** School of Mechanical Engineering, Nanjing University of Science and Technology, Nanjing 210094, China; czp@njust.edu.cn (Z.C.); yuanzhao@njust.edu.cn (Y.Z.); chuanhao.zhang@njust.edu.cn (C.Z.); hezhangz@njust.edu.cn (H.Z.)

**Keywords:** UAV, flight parameter identification, launch force

## Abstract

Flight parameters are crucial criteria for UAV control, playing a significant role in ensuring the safe and efficient completion of missions. Launch force and airspeed information are key parameters in the early and middle stages of flight, serving as important data for monitoring the UAV’s flight status. In response to challenges such as weak launch force, low identification rates, small airspeed, and low recognition accuracy in UAVs, a method for identifying UAV flight parameters based on launch force and airspeed is proposed. From the aspect of launch force identification, a recognition method based on a low-g value accelerometer information source is proposed, utilizing a ‘multi-level time window + threshold’ approach. For airspeed identification, an optimization method for airspeed measurement under the Kalman filter architecture is introduced. A device for airspeed measurement based on pressure sensors is designed, and the recommended installation position is determined through simulation. Furthermore, the feasibility and robustness of the proposed launch force identification and airspeed measurement optimization methods are validated through simulation. Finally, the effectiveness of the design is verified through centrifuge and wind tunnel experiments. This research provides technical support for the identification of the launch force and airspeed measurement in UAVs.

## 1. Introduction

In recent years, unmanned aerial vehicles (UAVs) have experienced rapid development in both civilian and military sectors [1,2,3]. UAVs implement control through sensitive flight parameters such as launch force, flight attitude, altitude, speed, position, etc., ensuring the safety and efficient completion of tasks [4,5,6,7]. The flight phases of a UAV can be divided into the initial stage, mid-stage, and final stage. The initial stage involves the UAV being launched through various means, such as taxiing, cannon firing, catapulting, and ejection. The mid-stage is characterized by the UAV’s cruising flight, while the final stage is when the UAV executes its mission. The most typical flight parameter in the initial stage is the launch force, which is used to determine whether the UAV has been launched. In the mid-stage, the airspeed information can be referenced to identify the UAV’s flight status. The final stage of the flight is completed based on the detected target information to fulfill the mission.

The launch force of UAVs is characterized by a low amplitude, a narrow pulse width, and difficulty in recognition. There is currently limited research on the identification of the launch force for UAVs. Zhang and colleagues proposed a new hydrodynamic catapult scheme for UAVs, analyzing the variation pattern of the UAV launch force and providing the launch force change curve [8]. Li employed a numerical analysis to study the dynamic characteristics of UAV ejection [9,10]. Prasad and others designed a UAV launch force mitigation system to reduce rearward impact [11]. Nelson and his team investigated the impact of the launch force on UAV launch vibration, designing mechanisms to improve the vibration characteristics of UAVs under recoil [12]. Butt and collaborators introduced a method to design launch frames based on the UAV launch force [13]. Zhang obtained the launch force curve of UAVs through simulation [14]. It can be observed that the current research on UAV launch force primarily focuses on guiding the design of the launch system using launch force, without studying the identification of the launch force from the perspective of UAVs to determine their flight status.

In the middle phase of UAV flight, the flight state is controlled based on the flight speed, specifically the airspeed value. Common methods for measuring the UAV airspeed include a pitot tube velocity measurement [15,16,17,18], integrated GPS with low-cost inertial navigation systems (IMU), and others [19,20,21,22]. For UAVs where precision in airspeed measurement is not crucial, the high cost of integrating GPS with IMU can be prohibitive. Additionally, pitot tubes may lead to sensor measurement errors due to issues, such as icing, dust, and water particle blockage [23,24]. Borup and colleagues proposed a machine learning method for estimating the air data parameters of a small fixed-wing UAV based on distributed pressure sensors, obtaining results such as the airspeed [25]. Callegari and others introduced a sensor data fusion strategy based on error propagation analysis, directly applying capacitive sensors to UAVs and measuring the airspeed through redundant pressure readings [26]. Fries and his team embedded pressure sensors and sensing elements into a flexible body, creating a compact, accurate, reliable, and low-power sensor for measuring the UAV airspeed [27]. From the synthesis of the above literature, it can be observed that small UAVs have strict requirements for the size, weight, power consumption, and cost of airspeed measurement systems. Traditional measurement methods are not suitable. Pressure sensors, on the other hand, exhibit characteristics such as low power consumption, small size, and low cost. Therefore, there is an urgent need to design an airspeed measurement system using low-cost, highly integrated pressure sensors to achieve a high-precision measurement of the UAV airspeed.

This paper focuses on UAVs and investigates a flight parameter identification method based on launch force and airspeed. In terms of launch force identification, a method using ‘multi-level time window + threshold’ is proposed, and through simulation, it is verified that this identification method exhibits good resistance to interference and high robustness. For airspeed identification, an optimization method for airspeed measurement is proposed based on the Kalman filter. A device for airspeed measurement is designed based on pressure sensors, and the recommended installation position is analyzed through simulation. The feasibility of launch force and airspeed identification methods is validated through centrifuge and wind tunnel experiments. The results indicate that the proposed identification methods in this paper have high reliability and strong resistance to interference, providing new insights into UAV flight parameter identification.

This paper is organized as follows. Section 2 introduces the ‘multi-level time window + threshold’ method for recognizing the launch force of unmanned aerial vehicles. Section 3 proposes a method for measuring the airspeed and designs an airspeed measurement device. Section 4 verifies the anti-interference of the ‘multi-level time window + threshold’ method and the reliability and recommended installation position of the airspeed measurement device through simulation. Section 5 validates the feasibility of the launch force and airspeed recognition methods through centrifuge tests and wind tunnel experiments. Finally, Section 6 provides a summary.

## 2. Method for Launch Force Identification

**Figure 1:**
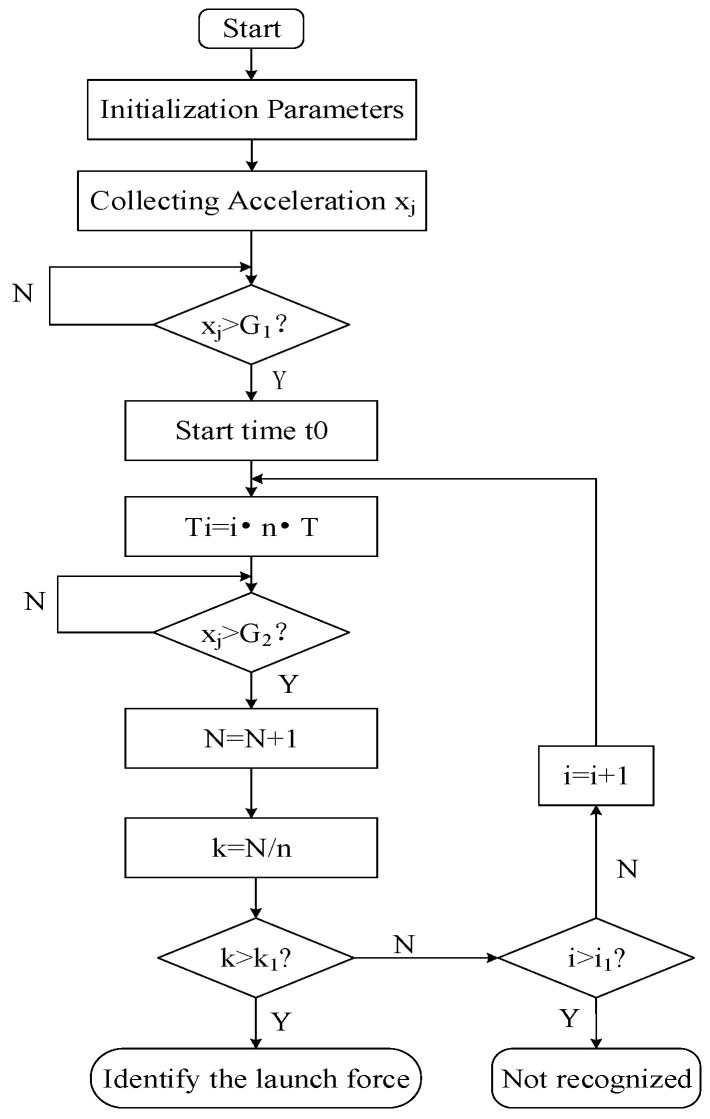
Flowchart of the ‘multi-level time window + threshold’ recognition method.

UAVs have a relatively low launch force, but the launch process inevitably introduces noise, resulting in a low signal-to-noise ratio. Common launch force identification methods include approaches, such as ‘threshold + sequence + time window’ [28], ‘single threshold + time window’, ‘double threshold + time window’, and ‘threshold + sliding time window’ [29]. These methods are primarily suitable for signals with large amplitudes, high signal-to-noise ratios, and low noise. However, they face challenges when applied to launch force signals, especially those with significant interference in the initial stage. In response to the aforementioned issues, this article proposes a ‘multi-level time window + threshold’ method for recognizing the launch force of unmanned aerial vehicles. It possesses the characteristic of good anti-interference. The process is illustrated in Figure 1.

**Figure 2:**
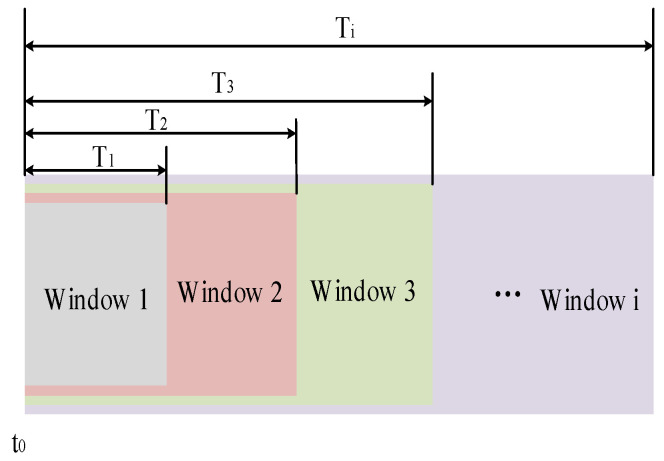
Schematic diagram of multi-level time window.

Before launching the UAV, set the parameters, such as the threshold values $G_{1}$ and $G_{2}$, number of sampled points within the first-level window (*n*), sampling period (*t*), window levels (*i*), proportional threshold ($k_{1}$), and count value (*N*). After the drone is launched, the low-g accelerometer sensor collects three-axis accelerations ($x_{j}$). When the acceleration along any axis exceeds $G_{1}$, record the timestamp ($t_{0}$) as the starting point for the multi-level window timing. The first-level window samples n points with a window time ($T_{1}$) of n·t; the second-level window samples 2n points, and so on. The ith-level window samples i·n points, and its duration $T_{i}=i\cdot n\cdot T$. The window diagram is illustrated in Figure 2. Starting from the first-level window, assess the number of instances where $x_{j}$ exceeds $G_{2}$ within each window. If the criteria are met, increment the count value (*N*); otherwise, keep *N* unchanged. At the end of each window, calculate the ratio (*k*) of *N* to the total sampled points within the window. Only when *k* exceeds the set ratio threshold ($k_{1}$) can the identification of the launch force be confirmed. If the requirements are not met within the specified *i*-level time window, the launch force is considered not recognized.

## 3. Method for Airspeed Identification

**Figure 3:**
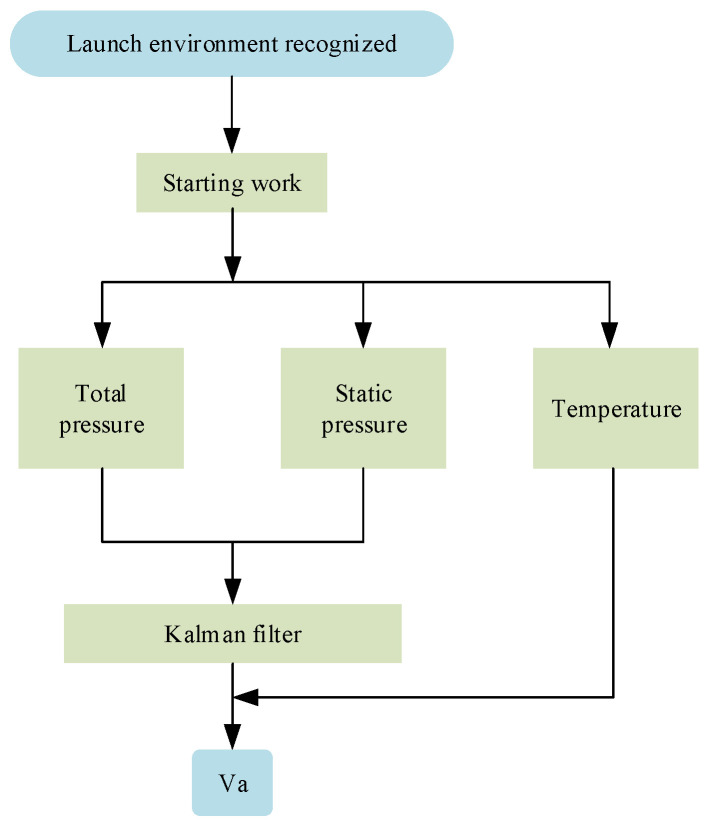
Schematic diagram of airspeed measurement.

The measurement of the UAV airspeed typically employs a pitot tube for airspeed measurement [16], but pitot tubes have drawbacks, such as a large size, susceptibility to blockage, and high cost [23,24]. This paper, based on the Bernoulli equation, utilizes a low-cost MEMS pressure sensor to design a UAV airspeed measurement device, measuring the total pressure, static pressure, and temperature values. During sensor measurements, the inevitable generation of noise was analyzed by Marinov using the Allan variance method to characterize the noise types of the MEMS pressure sensors [30]. The results indicate that random walk or white noise predominates. Typically, a Kalman filter is chosen for the noise filtering of sensors. The Kalman filter is an effective autoregressive filter and an optimal recursive mathematical processing method. It can predict and estimate the current system state of a linear dynamic system under a series of incomplete and Gaussian noise measurements [31]. Therefore, in this paper, Kalman filtering is also employed to filter the pressure values, obtaining more accurate airspeed values. The Kalman filtering algorithm, as shown in Algorithm 1, takes the sensor-measured total pressure and static pressure data as observation values ($z_{k}$) and calculates the precise air pressure. Considering the relatively small temperature error in the sensor measurements, the measured temperature values are treated as true values. Based on the filtered total pressure, static pressure, and temperature data, the airspeed of the unmanned aerial vehicle is calculated. The airspeed measurement process is illustrated in Figure 3.
**Algorithm 1:** Kalman Filtering Algorithm.1 Parameter Initialization: ${\hat{x}}_{0}^{-}$, $P_{0}^{-}$, ${\hat{x}}_{0}$, $P_{0}$2 State Prediction Equation:$$\;{\hat{x}}_{k}^{-}=f\left({\hat{x}}_{k-1},u_{k-1},0\right)$$3 Calculate Error Covariance: $P_{k}^{-}=A_{k}P_{k-1}A_{k}^{T}+W_{k}Q_{k-1}W_{k}^{T}$4 Filter Gain: $K_{k}=P_{k}^{-}H_{k}^{T}{\left(H_{k}P_{k}^{-}H_{k}^{T}+V_{k}R_{k}V_{k}^{T}\right)}^{-1}$5 State Estimation:${\hat{x}}_{k}={\hat{x}}_{k}^{-}+K_{k}\left(z_{k}-h\left({\hat{x}}_{k}^{-},0\right)\right)$6 Estimate Error Covariance: $P_{k}=\left(I-K_{k}H_{k}\right)P_{k}^{-}$

### Theoretical Model

According to the Bernoulli equation, the pressure relationship for a UAV in flight is [32]:

(1) $$p_{t}=p_{d}+p_{s}$$

In Equation (1), $p_{t}$ represents the total pressure, which is the pressure measured perpendicular to the direction of the airflow. $p_{d}$ is the dynamic pressure, indicating the pressure when the airflow velocity is zero. $p_{s}$ is the static pressure, representing the pressure exerted on the surface when the object is at rest or moving at a constant speed in a straight line. The airspeed of the UAV is given by [33]:

(2) $$v_{UAV}=K\sqrt{\frac{2(p_{t}-p_{s})}{\rho_{s}}}=K\sqrt{\frac{2p_{d}}{\rho_{s}}}$$

where *K* is the calibration factor determined during the pitot tube calibration to account for sensitivity to the air temperature and pressure. $\rho_{s}$ represents the air density, and its calculation is given by [34]:

(3) $$\rho =\frac{pM_{a}}{ZRT}\left[1-x_{v}\left(1-\frac{M_{v}}{M_{a}}\right)\right]$$

In Equation (3), $M_{a}$ is the molar mass of dry air, *Z* is the air compressibility factor, *R* is the molar gas constant, *T* is the thermodynamic temperature of the air, $x_{v}$ is the molar fraction of water vapor, and $M_{v}$ is the molar mass of water.

Substituting Equation (3) into Equation (2), we obtain:

(4) $$v_{UAV}=\frac{\sqrt{2ZR}K}{\sqrt{M_{a}\left[1-x_{v}\left(1-\frac{M_{v}}{M_{a}}\right)\right]}}\sqrt{\frac{T\left(p_{t}-p_{s}\right)}{p_{s}}}$$

It can be observed that the airspeed value is related to the total pressure, static pressure, and temperature. The airspeed measurement device calculates the airspeed by measuring these three values.

#### Structural Model of Airspeed Measurement Device

**Figure 4:**
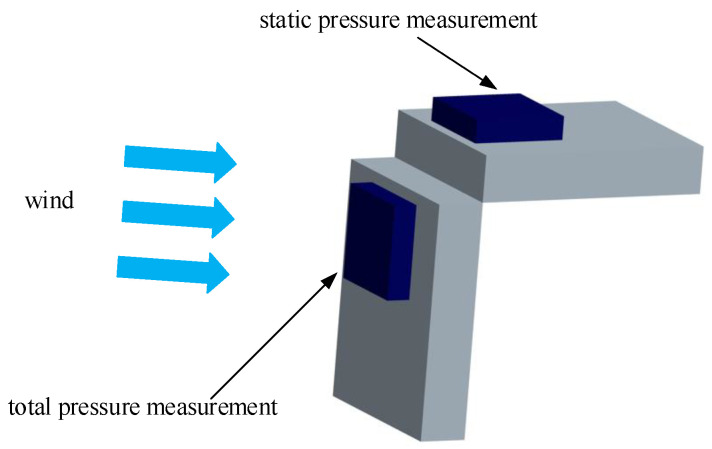
Schematic diagram of the airspeed measurement device structure.

**Figure 5:**
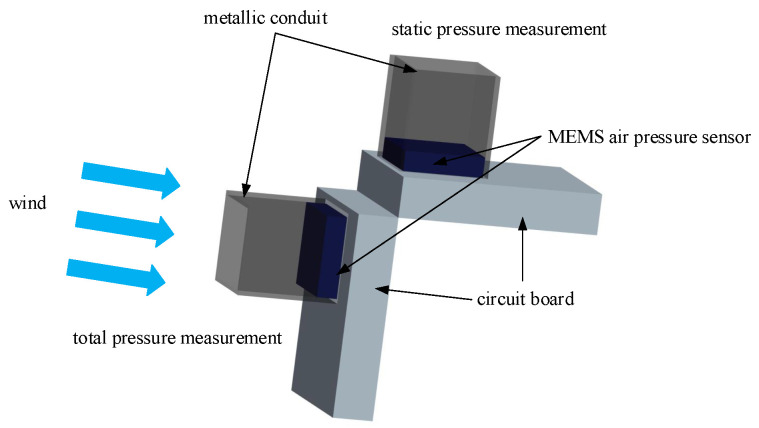
Schematic diagram of the improved structure of the airspeed measurement device.

**Figure 6:**
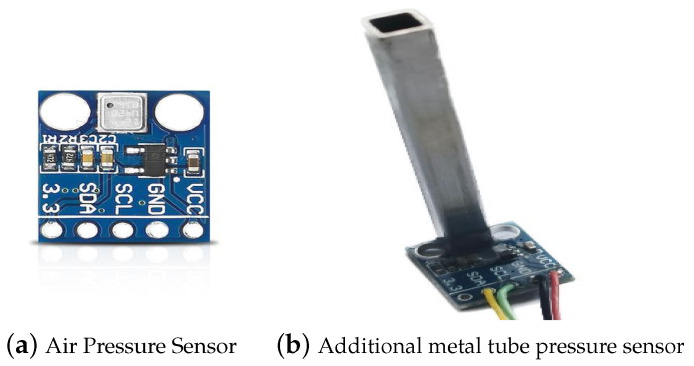
Comparison diagram of pressure sensor with and without added metal pipe.

The airspeed measurement device includes two vertically positioned air pressure sensors. One sensor is aligned with the direction of the unmanned aerial vehicle’s flight to measure the total pressure. The other sensor is installed vertically to the flight direction to measure the static pressure and temperature. The schematic diagram of the airspeed measurement device structure is shown in Figure 4. Inspired by references [27,35,36], additional metal pipes are attached to the outside of the pressure sensors to reduce the impact of the UAV body on the airflow field, allowing for more accurate pressure measurements. The improved structure is illustrated in Figure 5, and a physical representation is depicted in Figure 6.

## 4. Simulation Verification

### 4.1. Simulation Verification of the ‘Multi-Level Time Window + Threshold’ Recognition Method

**Figure 7:**
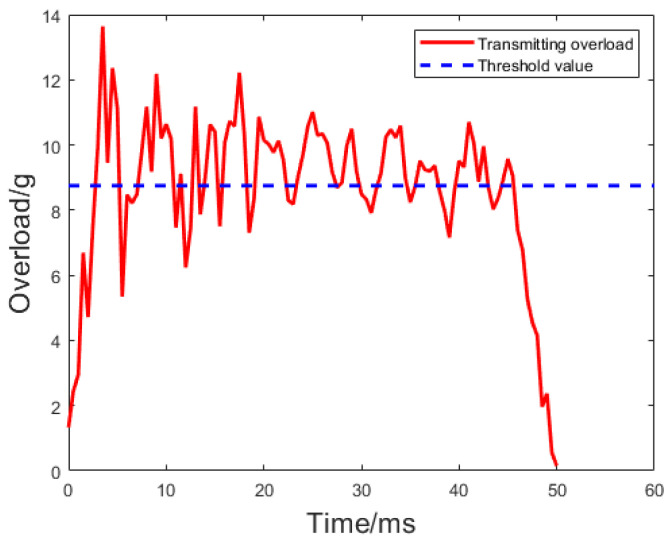
Fit launch force curve and threshold for UAV.

Referring to the UAV launch force curve in the literature [37,38], considering the inevitable noise generated by sensors during data acquisition, Gaussian white noise with zero variance is added to the curve to obtain a fitted launch force curve. When using the ‘threshold + time window’ method for environmental recognition, it is common to choose three-quarters of the maximum amplitude as the threshold [39]. Using this threshold, the fitted launch force curve and threshold are shown in Figure 7.

The launch force signals were identified using three different methods: the ‘multi-level time window + threshold’ recognition method, the ‘threshold + time window’ recognition method from reference [28], and the ‘threshold + sliding time window’ recognition method from reference [29]. The recognition results of the three methods are shown in Table 1. Algorithm 1 corresponds to the ‘multi-level time window + threshold’ method, Algorithm 2 corresponds to the ‘threshold + time window’ method, and Algorithm 3 corresponds to the ‘threshold + sliding time window’ method.

Algorithms 1 and 3 successfully identify the emission force signals, whereas Algorithm 2 fails to do so. This is because the ‘threshold + time window’ method, typically used to recognize signals with larger amplitudes, involves setting a higher threshold to minimize interference signals. This method is reliable in scenarios with significant force amplitudes and minimal interference. However, in the case of low-emission force signals from UAVs, both the amplitude and threshold are small. Interference signals can cause signals within the time window exceeding the threshold not to meet the requirements, leading to the failure of force recognition. For the UAV emission force signals in the graph, when using the ‘threshold + time window’ method, early signals exhibit significant noise ($k<k_{1}$), failing to meet the threshold requirement and resulting in recognition failure. In the ‘multi-level time window + threshold’ method, initial levels of time windows may not recognize the force signals, but as the window widens, the noise gradually decreases, N increases ($k>k_{1}$), and the emission force becomes recognizable.

**Figure 8:**
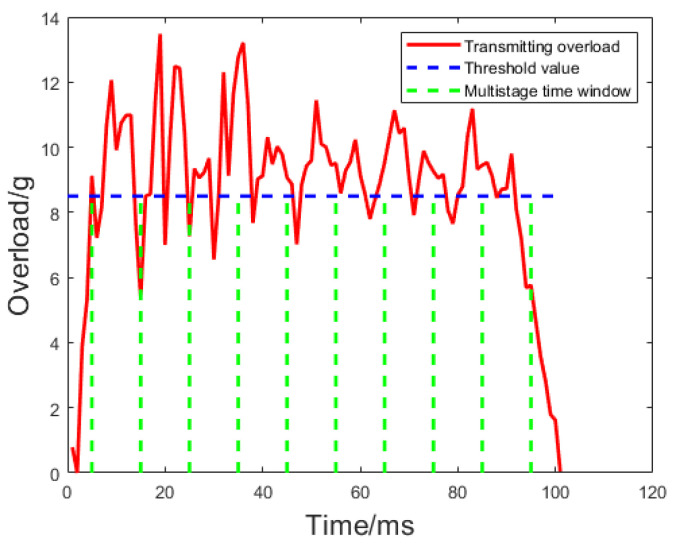
Recognition result of ‘multi-level time window + threshold’.

The recognition results of the proposed ‘multi-level time window + threshold’ method are shown in Figure 8, and the result data are presented in Table 2, with a set threshold $k_{1}$ = 0.8. It can be observed that there is significant noise in the early part of the signal. Consequently, the proportion does not meet the requirements within the first three windows, leading to the inability to recognize the launch force. As the signal noise gradually diminishes, the launch force is recognized within the fourth window. The recognition method proposed in this paper is suitable for scenarios with high noise. As the window widens, the number of points exceeding the threshold gradually increases, and the launch force stabilizes, making the recognition results more reliable.

**Figure 9:**
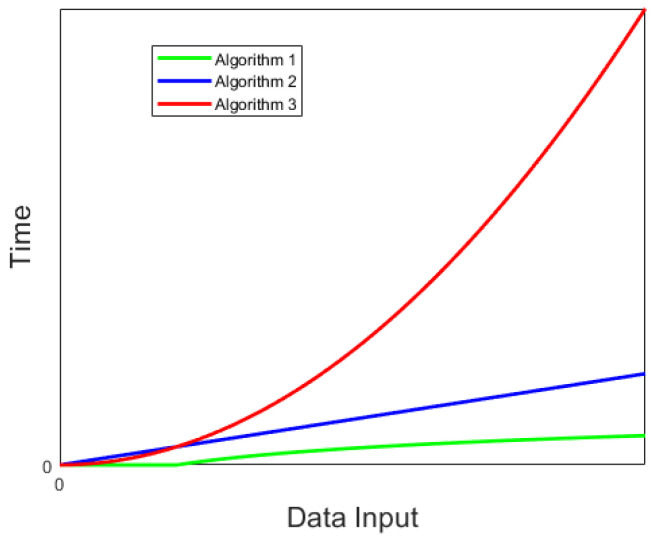
Three algorithm complexities comparison chart.

To compare the complexity of the three algorithms, an analysis was conducted using the Big-O notation method proposed by Juris Hartmanis and Richard E. Stearns [40,41,42]. In Big-O notation, O represents the order of magnitude, and O(f(n)) indicates how the algorithm’s complexity grows as the size of the problem (n) increases. The complexities of the three algorithms, calculated using the Big-O notation method, are presented in Table 3. The runtime graphs of the algorithms are illustrated in Figure 9.

As can be seen, with the increase in the size of the problem, the time required for Algorithm 3 grows exponentially, indicating a high level of complexity. Algorithm 2 exhibits a linear relationship, suggesting a slightly higher complexity, while Algorithm 1 shows an exponential increase with the lowest complexity. Therefore, the proposed ‘multi-level time window + threshold’ recognition method in this study has low complexity, high reliability, strong interference resistance, and is suitable for UAV emission force recognition.

### 4.2. Simulation Validation of Aerodynamic Characteristics for Airspeed Measurement Device

**Figure 10:**
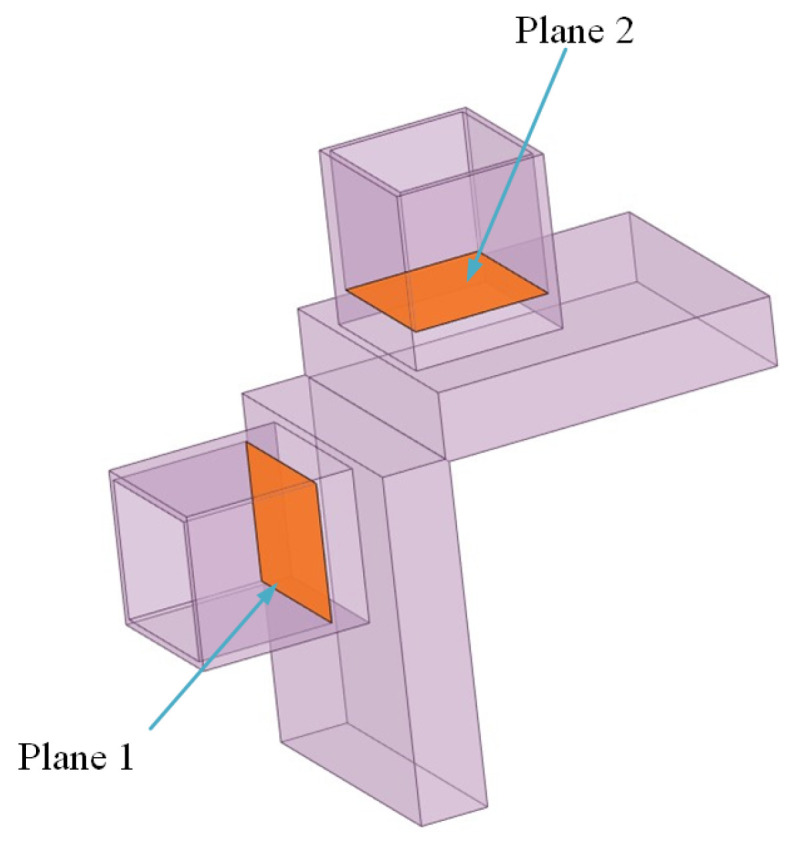
Airspeed measuring device simulation structure diagram.

Based on the airspeed measurement device designed in this paper, a model is established for simulation using ANSYS Fluent software 6.0 to analyze the feasibility of the airspeed measurement device, and the simulation parameters are set as shown in Table 4. The model is depicted in Figure 10, where plane 1 represents the total pressure surface, plane 2 is the static pressure surface, and the wind direction is perpendicular to plane 1 from left to right. The sensors measure the changes in air pressure on plane 1 and plane 2, as well as the temperature in plane 2.

**Figure 11:**
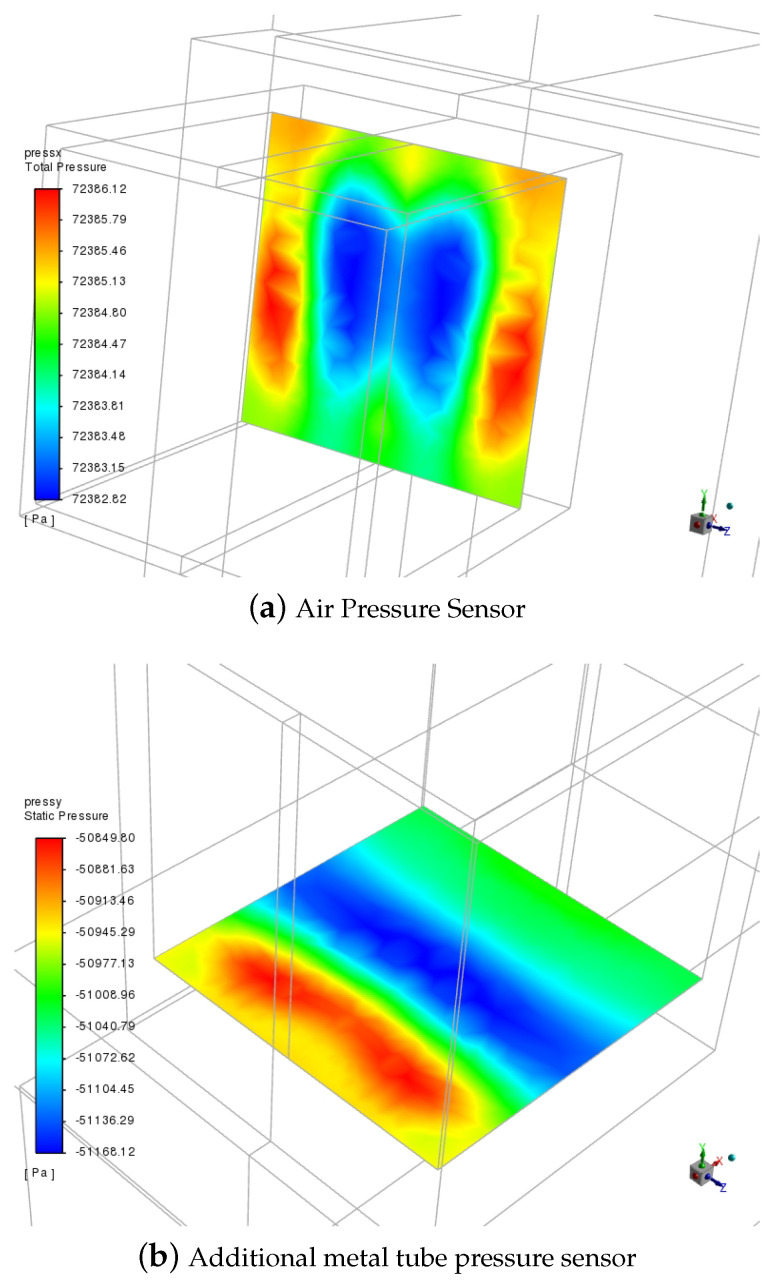
The air pressure distribution on plane 1 and plane 2.

The simulation conditions were set with wind speeds of 0.3 Ma, 0.5 Ma, 0.7 Ma, and 0.9 Ma. Taking the condition with the most significant results, the wind speed of 0.9 Ma, as an example, the pressure distribution on both plane 1 and plane 2 is shown in Figure 11 where the color intensity increases with higher pressure. It can be observed that the pressure is minimal and most uniformly distributed in the middle sections of both plane 1 and plane 2. In contrast, the pressure around the periphery is more scattered. This indicates that the pressure is smallest and most uniform in the middle sections, while the pressure distribution around the periphery is larger. This non-uniformity may lead to inaccurate air pressure measurements. Therefore, it is recommended that sensors measure the pressure in the middle sections of both planes to enhance the measurement accuracy.

**Figure 12:**
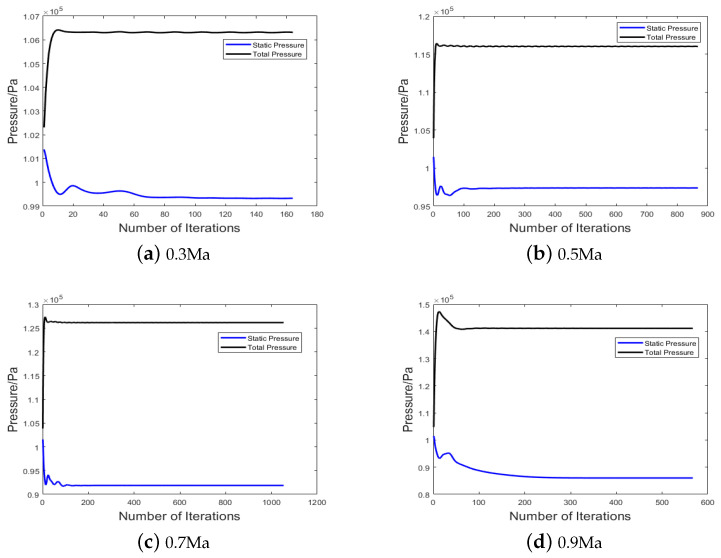
Curves of total pressure and static pressure for four operating conditions.

The total pressure and static pressure curves for the four operating conditions are shown in Figure 12. Using the measurement results combined with Equation (5), the airspeed values are calculated and compared with the simulated conditions to verify the accuracy of the simulation results. The results are presented in Table 5. The formula for calculating the simulation result error is as follows, where $k_{1}$ is the set simulation wind speed, and $k_{2}$ is the airspeed calculated based on the simulated results of the total pressure, static pressure, and temperature, with the numerical values stabilized after multiple iterations.

(5) $$\delta =\frac{k_{2}-k_{1}}{k_{1}}\times 100\%$$

It can be observed that, under the four operating conditions, the error between the simulated results and the set conditions is around 10%, indicating the accuracy of the simulation results. As the wind speed increases, the error gradually rises. This is attributed to the gradual increase in the total pressure and the simultaneous decrease in the static pressure, leading to larger amplitude changes and an accumulation of errors.

In summary, the simulation results demonstrate that the airspeed measurement device designed in this paper can effectively identify the flight environment during the flight process with high accuracy.

### 4.3. Simulation Verification of the UAV’s External Aerodynamic Characteristics

The airspeed measurement device needs to be installed on the surface of the UAV, and the appropriate installation position is crucial for the accuracy of data collection. In this section, an external aerodynamic simulation is conducted for the UAV to analyze the pressure distribution at various positions during flight. Based on the simulation results, the optimal sensor installation position is determined.

**Figure 13:**
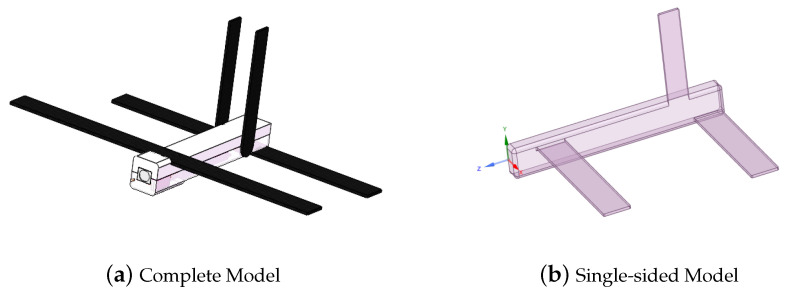
UAV simulation model.

A folding-wing UAV model is selected, as depicted in Figure 13. The model is symmetrical, and to minimize the grid partitioning complexity during the simulation process, an aerodynamic simulation analysis is conducted on one side of the UAV.

**Figure 14:**
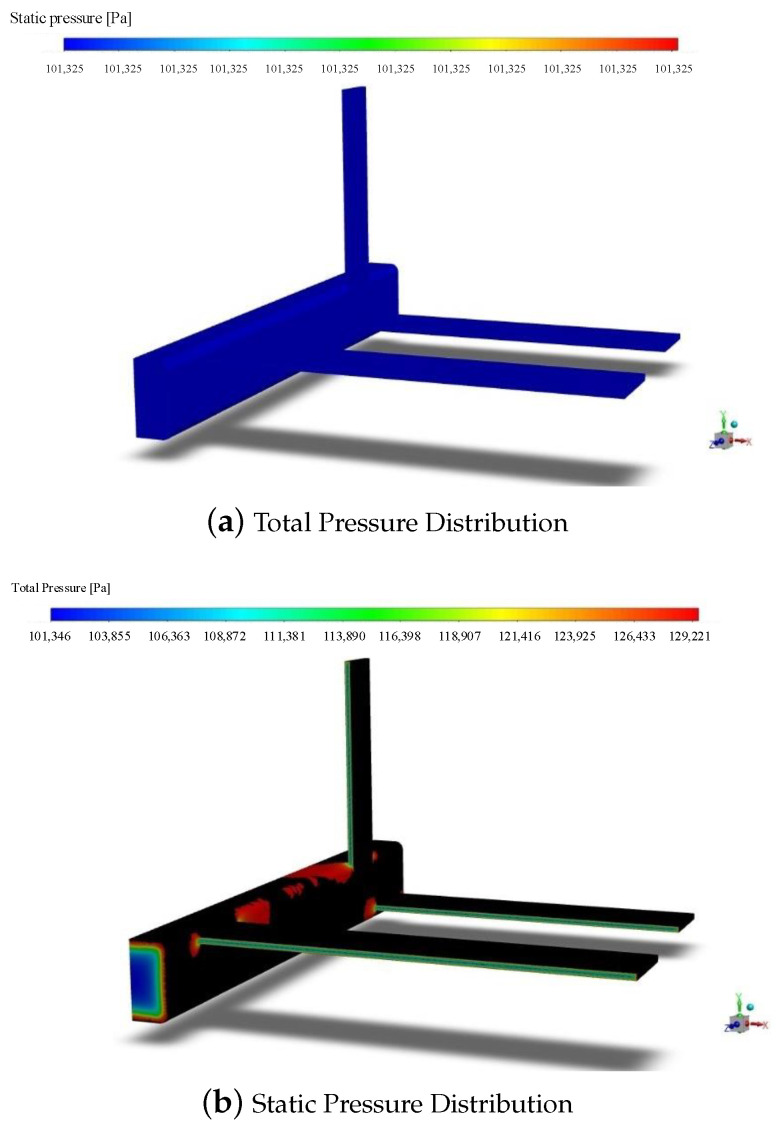
Pressure distribution map of the UAV.

The simulation parameters for the UAV are set as shown in Table 6, and the results are shown in Figure 14. It can be observed that the static pressure is the same at various positions on the UAV. The total pressure values on the windward surfaces of the front, leading edge of the wing and the trailing edge of the wing are relatively high. Considering the convenience of installation and stability of measurements, the optimal installation position for the airspeed measurement device is on the windward surfaces of the front and leading edge of the wing of the UAV.

## 5. Verification of the Launch Force Identification Method

To validate the feasibility of the proposed UAV launch force and airspeed identification methods in this paper, a centrifuge experiment was conducted to verify the launch force identification method, and a wind tunnel experiment was performed to validate the airspeed identification accuracy.

### 5.1. Simulation Verification of the UAV’s External Aerodynamic Characteristics

**Figure 15:**
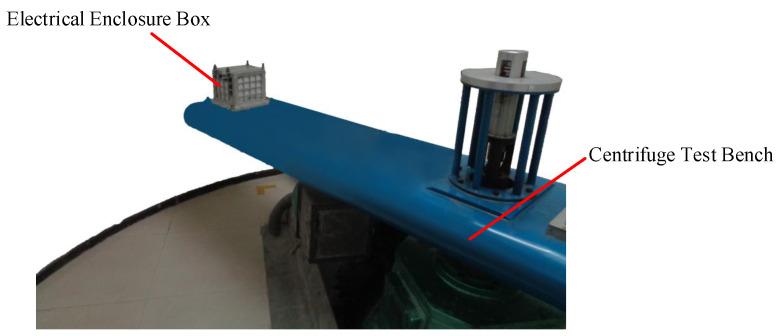
Centrifuge test diagram.

**Figure 16:**
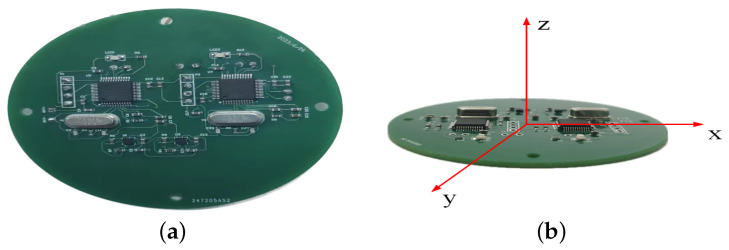
Circuit diagram. (**a**) Sensor and control circuit diagram. (**b**) Circuit three-axis direction schematic diagram.

A centrifuge experiment was conducted to simulate the launch force of the UAV, as shown in Figure 15. The circuit containing the accelerometer sensor was installed inside the centrifuge test bed’s fixed box, aligning the sensor’s Z-axis direction parallel to the test bed’s rocker arm for simulating the UAV launch force. The centrifuge force magnitude was controlled by adjusting the rotation speed of the test bed. The sensor and control circuit are depicted in Figure 16a, and the schematic diagram of the circuit’s three-axis directions is shown in Figure 16b.

**Figure 17:**
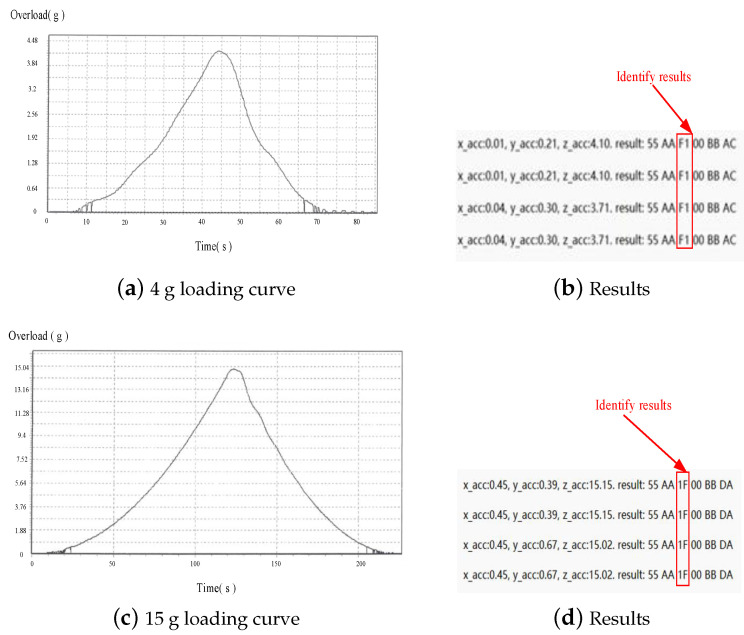
Launch environment test results.

The measured centrifuge force values were transmitted to the upper computer to monitor whether the control module recognized the launch environment. The launch force recognition range was set at 5–15 g. The centrifuge test bed was loaded with centrifuge forces of 4 g and 15 g separately to verify whether the UAV could recognize them. The loading curves of the centrifuge test bed and the UAV recognition results are shown in Figure 17.

In the experimental results, the flag F1 indicates the failure to recognize the launch force, while 1F indicates the recognition of the launch force. It can be observed that when the centrifuge test bed is loaded with a centrifuge force of 4 g, the maximum overload collected by the sensor is 4.1 g, with an error of 2.5%, and the UAV fails to recognize the launch force. When loading the environment with a force of 15 g, the collected overload is 15.15 g, with an error of 1%, and the UAV can recognize the launch force.

The experiments demonstrate that the UAV launch force recognition module designed in this paper can accurately identify the launch force. The proposed ‘multi-level time window + threshold’ method can effectively recognize the environment with good anti-interference performance, providing a new approach for UAV launch force recognition.

### 5.2. Verification of the Airspeed Identification Method

To assess the effectiveness of the airspeed identification method, a wind tunnel experiment was conducted. The wind tunnel was configured with a known wind speed as the true airspeed. The measured airspeed values, the calculated airspeed after Kalman filtering, and the wind tunnel setting were compared to evaluate the accuracy of the airspeed measurement device and the filtering effect of the Kalman filter.

**Figure 18:**
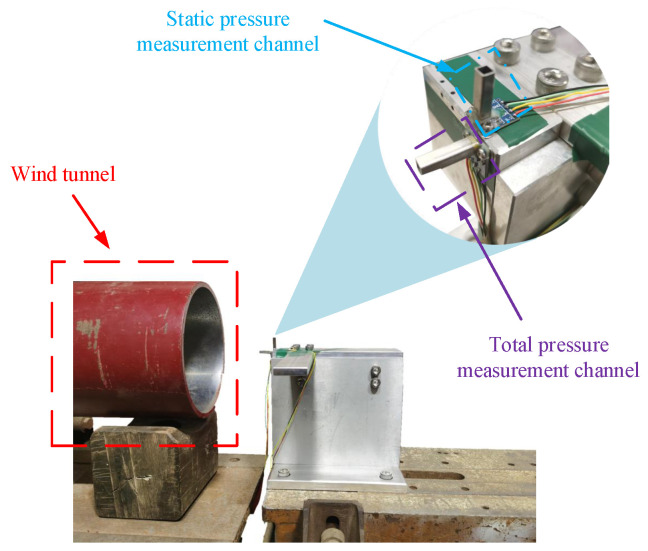
Wind tunnel test diagram.

**Figure 19:**
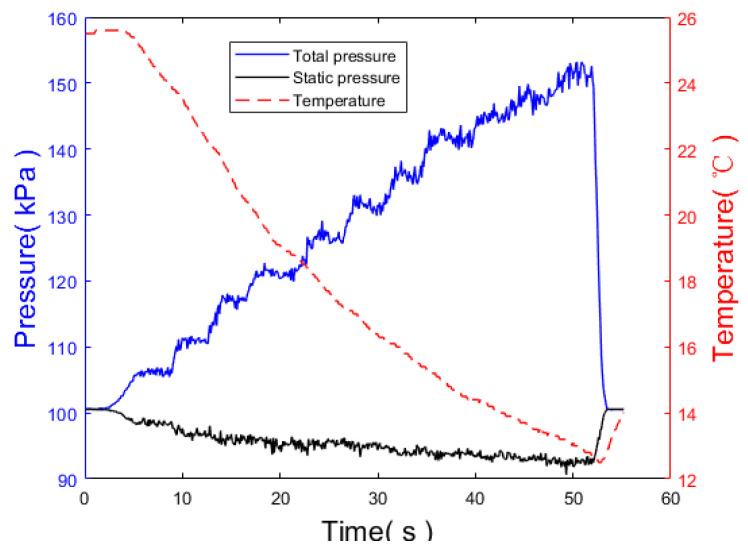
Changes in air pressure and temperature in wind tunnel test.

As shown in Figure 18, two pressure sensors are vertically installed. The wind tunnel is situated on the left side, and the airflow direction is toward the right. The total pressure measurement channel is directly facing the airflow direction, while the static pressure measurement channel is perpendicular to the airflow direction. The wind tunnel wind speeds are set at various levels, ranging from low to high, with each wind speed level maintained for a certain period. The measured data for the total pressure, static pressure, and temperature are depicted in Figure 19.

It can be observed that with the increase in the wind tunnel wind speed, the total pressure gradually increases with a significant amplitude, while the static pressure gradually decreases but with a weaker amplitude. The total pressure variation is most sensitive to changes in wind speed, while the static pressure is less affected by changes in wind speed, consistent with the simulation results.

In the literature [1,43], the Root Mean Square Error (RMSE) is commonly employed to assess the performance of filters. The RMSE represents the deviation between observed values and true values, with smaller values indicating better filtering effectiveness.

(6) $$\mathrm{RMSE}=\sqrt{\frac{1}{N}\sum_{i=1}^{N}{\left(x_{m}\left(i\right)-x_{c}\left(i\right)\right)}^{2}}$$

In the equation, $x_{m}\left(i\right)$ is used to measure the amplitude of the signal, $x_{c}\left(i\right)$ is employed to calculate the signal amplitude, and N represents the length of the signal.

**Figure 20:**
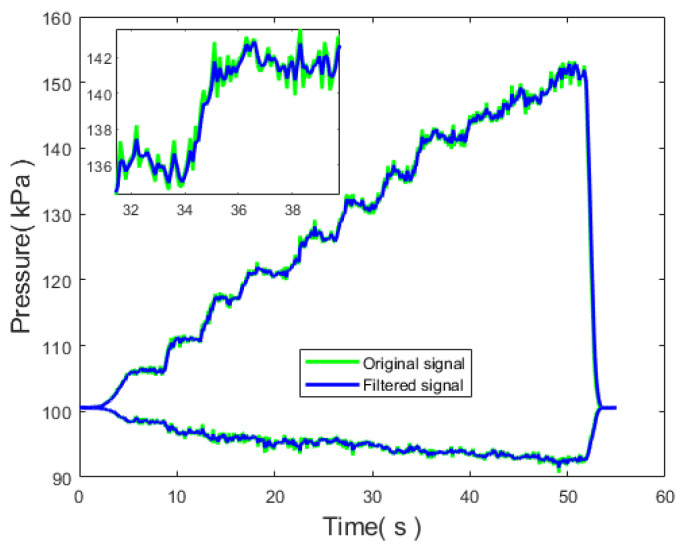
Comparison of pressure values before and after filtering.

The data after filtering with the Kalman filter for the total pressure and static pressure are shown in Figure 20. The comparison of the RMSE between the original signal and the filtered signal is presented in Table 7.

For the significantly fluctuating total pressure signal, the RMSE decreased by 18.26%, indicating a significant reduction in error and a noticeable improvement in filtering effectiveness.

(7) $${\mathrm{RMSE}}^{\prime }=\sqrt{\frac{1}{N}\sum_{i=1}^{N}{\left(v_{1}\left(i\right)-v_{2}\left(i\right)\right)}^{2}}$$

(8) $${\mathrm{RMSE}}^{\prime \prime }=\sqrt{\frac{1}{N}\sum_{i=1}^{N}{\left(v_{3}\left(i\right)-v_{2}\left(i\right)\right)}^{2}}$$

In the equation, $v_{1}\left(i\right)$ represents the measured airspeed, $v_{2}\left(i\right)$ is the true airspeed, and $v_{3}\left(i\right)$ is used to calculate the airspeed value. To compare the filtering effects, the RMSE error rate is defined by the formula as follows, and the results are presented in Table 8.

(9) $$\varepsilon_{\mathrm{RMSE}}=\frac{{\mathrm{RMSE}}^{\prime }-{\mathrm{RMSE}}^{\prime \prime }}{{\mathrm{RMSE}}^{\prime }}\times 100\%$$

**Figure 21:**
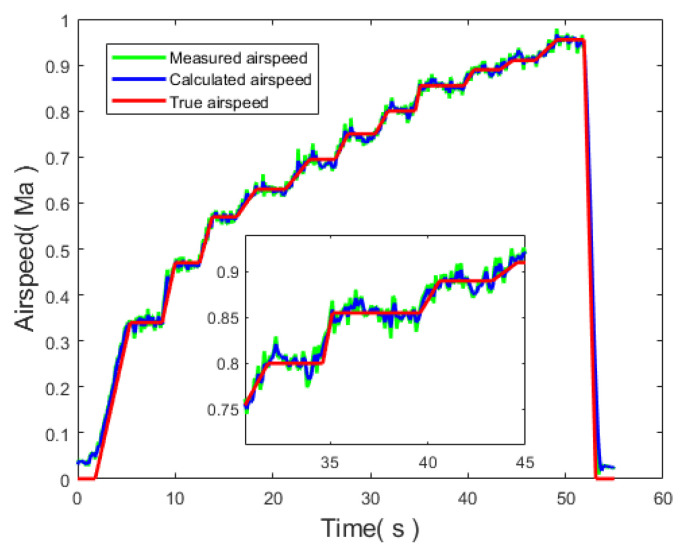
Comparison of measured airspeed, calculated airspeed, and true airspeed.

According to Figure 21 and Table 8, it can be observed that the airspeed measurement device designed in this study is capable of measuring airspeed values. By applying a Kalman filter to filter the pressure data, the calculated airspeed values are closer to the actual values, resulting in a significant reduction of 26.42% in error. Utilizing this method allows for the accurate identification of the UAV airspeed, thereby enhancing the precision of unmanned aerial vehicle flight parameter recognition.

## 6. Conclusions

This paper investigates launch force and airspeed identification methods for UAVs. For launch force identification, a ’multi-level time window + threshold’ method is proposed based on the information source from low-g value accelerometer sensors. The simulation results indicate to good anti-interference performance of this method. Regarding airspeed identification, an optimization method for airspeed measurement under the Kalman filter architecture is proposed. A design for an airspeed measurement device based on pressure sensors is presented, with the recommended installation position determined through simulation. Finally, the effectiveness of the identification methods is verified through centrifuge and wind tunnel experiments. This research provides technical support for launch force and airspeed identification in UAVs.

## Figures and Tables

**Table 1:** Comparison table of recognition results for three algorithms.

Algorithm	Recognition Results
Algorithm 1	Yes
Algorithm 2	No
Algorithm 3	Yes

**Table 2:** ‘Multi-level time window + threshold’ recognition result table.

Window Index	Data Volume Greater than Threshold	Total Data	Ratio *k*	Recognition Results
1	7	100	0.70	No
2	14	200	0.70	No
3	22	300	0.73	No
4	32	400	0.8	Yes

**Table 3:** Comparison table of algorithm complexities.

Algorithm	Big-O Notation
Algorithm 1	O(log n)
Algorithm 2	O(n)
Algorithm 3	O(n2)

**Table 4:** Airspeed measurement device parameters.

Name	Dimensions
Sensor	0.5 cm ∗ 0.5 cm
Metallic conduit	5 cm
Circuit board	3 cm ∗ 2.5 cm

**Table 5:** Simulation results for four operating conditions.

$k_{1}$/Ma	$P_{t}$/Pa	$P_{s}$/Pa	T/K	$k_{2}$/Ma	δ
0.3	106,323	99,330	302	0.33	10.0%
0.5	116,015	97,389	310	0.55	10.0%
0.7	126,139	91,873	319	0.78	11.4%
0.9	141,136	86,015	329	1.04	15.6%

**Table 6:** UAV simulation setup parameters.

Atmospheric Pressure	101,325 Pa
Temperature	300 K
Angle of Attack	1°
Velocity	0.6 Ma

**Table 7:** Comparison of RMSE between original and filtered signals.

	Total Pressure	Static Pressure
Original signal	16.54	2.40
Filtered signal	13.52	1.87

**Table 8:** Comparison table of filtering effects.

RMSE’	RMSE’’	$\varepsilon_{\mathrm{RMSE}}$
0.0159	0.0117	26.42

## Data Availability

The data presented in this study are available on request from the corresponding author.
